# The effect of remote peer support on stigma in patients after breast cancer surgery during the COVID-19 pandemic: A protocol for systematic review and meta-analysis

**DOI:** 10.1097/MD.0000000000026332

**Published:** 2021-06-18

**Authors:** Dandan Liang, Ruiying Jia, Jingfen Yu, Zhen Wu, Chaoran Chen, Guangli Lu

**Affiliations:** a Institute of Nursing and Health, College of Nursing and Health; b Institute of Business, School of Business, Henan University, Jinming Avenue, Kaifeng, Henan, China.

**Keywords:** breast cancer, coronavirus disease 2019, meta-analysis, protocol, remote peer support, stigma, systematic review

## Abstract

**Background:**

Patients after breast cancer surgery have a high sense of stigma due to the formation of surgical scars, loss of breast shape or other reasons, leading to anxiety, depression, and other adverse mental health problems, thus reducing their quality of life. Remote peer support intervention based on telephone, internet or email is low-cost and easy to spread, and protects patients’ privacy, solves the barriers to access that many patients face when attending face-to-face programs. Therefore, remote peer support may be an effective way to reduce stigma and improve mental health in patients after breast cancer surgery during the coronavirus disease 2019 (COVID-19) pandemic.

**Methods:**

Eight databases (PubMed, Embase, Cochrane Library, CNKI, PsycNET, MEDLINE, Psychology & Behavioral Sciences Collection and Web of Science) will be used to select eligible studies that were published from inception to May, 2021. The eligible studies will be screened, extracted and then the methodological quality will be evaluated independently by 2 reviewers. Review manager software version 5.3 software and Stata version 14.0 software will be used for meta-analysis.

**Results:**

The results of this study will show the effect of remote peer support on stigma, depression and anxiety in patients after breast cancer surgery during the COVID-19 pandemic and will be submitted to a peer-reviewed journal for publication.

**Conclusion:**

The results of this study will provide evidence for the effectiveness of remote peer support in patients after breast cancer surgery during the COVID-19 pandemic.

**PROSPERO registration number:**

CRD42021255971.

## 1 Introduction

Breast cancer is a serious public health problem and a leading cause of cancer death among women.^[1–3]^ According to the statistics from the World Health Organization (WHO), breast cancer accounts for 11.6% of all types of cancers and 6.6% of global mortality.^[4]^ In modern society, the treatment of breast cancer has entered the era of multidisciplinary comprehensive treatment guided by biological characteristics, including molecular targeted therapy, hormone therapy, chemotherapy, radiotherapy, traditional Chinese medicine therapy, gene therapy, stem-cell therapy, surgery, and so on,^[5,6]^ among them, surgery is still the preferred method for patients with breast cancer, except for some special patients (poor general condition, serious diseases in main organs, or the elderly are weak and intolerable, etc).^[6]^ However, breast excision or scar formation caused by surgery will damage the integrity and functionality of the body, loss of self-image and sexual dysfunction of the patients, and then produce a high sense of stigma.^[7–9]^

Stigma refers to the feeling of isolation, rejection, degradation and criticism during a social process or personal experience which influences the outcomes of physical, psychological and social adjustment.^[10,11]^ Stigma is a major obstacle to postoperative care for patients with breast cancer,^[12]^ and serious stigma will lead to anxiety, depression or other adverse mental health problems, and then affect their quality of life.^[13]^ It has negative effects throughout the whole process from diagnosis to the end of treatment,^[12]^ and to a certain extent, it will hinder patients from actively seeking medical treatment, making them to conceal their conditions and delay treatment.^[14]^ Especially during the coronavirus disease 2019 (COVID-19) pandemic, quarantine and lockdown measures were often used to control the spread of the epidemic, which also limited people's activities to a certain extent,^[15]^ making it more difficult to address the stigma of patients after breast cancer surgery face to face. Therefore, it is necessary to find an effective solution.

Peer support refers to a variety of forms that enable people with similar diseases, physical conditions, or experiences to provide substantive help and emotional support to each other,^[16]^ building non-hierarchical reciprocal relationships by sharing similar life experiences with others, the more homogeneous peers, the more likely this support is to lead to understanding, empathy, and mutual help.^[17]^ At present, there are seven successful models of peer support intervention including professional-led group visits with peer exchange; peer-led face-to-face self-management programs; peer coaches; community health workers; support groups; telephone-based peer support; web- and e-mail-based programs.^[18]^ Among them, remote peer support interventions (telephone or web-based peer support) may be more promising than face-to-face interventions. Because they are low-cost and easy to spread, and they address the access barriers faced by many patients when attending face-to-face programs, and many patients prefer relatively anonymous and private forms of communication.^[18,19]^ Therefore, in the context of the COVID-19 pandemic, when face-to-face peer support intervention is limited to a certain extent, remote peer support may be more able to play its unique advantages and make up for the inconvenience brought by the epidemic to the treatment of stigma in patients after breast cancer surgery.

At present, there have been some studies on the effects of remote peer support on mental health problems in people with type 2 diabetes, heart disease, breast cancer, postpartum women, etc.^[20–23]^ However, there is no meta-analysis or systematic review on remote peer support intervention to improve stigma in patients after breast cancer surgery. Therefore, in order to find an effective way to reduce stigma in patients after breast cancer surgery during the COVID-19 epidemic, this study conducted a meta-analysis of randomized controlled trials on the improvement of stigma in patients after breast cancer surgery based on remote peer support intervention.

## 2 Methods and analysis

### 2.1 Study registration

This systematic review has been registered in PROSPERO CRD42021255971. We strictly abide by the Preferred Reporting Items for Systematic Review and Meta-Analysis Protocols (PRISMA-P) guidelines.^[24]^

### 2.2 Inclusion criteria for study selection

#### 2.2.1 Types of studies

This study will include randomized controlled trials investigating the effect of remote peer support on patients after breast cancer surgery during the COVID-19 pandemic. Duplicate research reports or insufficient data will be excluded.

#### 2.2.2 Types of participants

Patients after breast cancer surgery during the COVID-19 pandemic will be included, regardless of age, gender, educational status or racial restrictions. People with other serious physical diseases (such as stroke, myocardial infarction, or other malignant tumors) will be excluded.

#### 2.2.3 Types of interventions

In the intervention group, participants received remote peer support based on telephone, internet or email, or in combination with usual care, but peer support intervention are led by non-professionals. The control group included participants who accepted usual care, usual education, etc.

#### 2.2.4 Outcome indicators

The primary outcomes of the study are stigma, the secondary outcome will be depression and anxiety.

#### 2.2.5 Language

Published in English or Chinese.

### 2.3 Search strategy

The following electronic databases were utilized for selecting eligible studies published from inception to May 2021: PubMed, Embase, Cochrane Library, CNKI, PsycNET, MEDLINE, Psychology & Behavioral Sciences Collection and Web of Science. The search strategy in PubMed is as follows:

- #1 Search: (((((peer support[Title/Abstract]) OR (peer education[Title/Abstract])) OR (peer-led[Title/Abstract])) OR (peer coach[Title/Abstract])) OR (peer counsel[Title/Abstract])) OR (peer mentor[Title/Abstract]) Filters: Abstract Sort by: Publication Date
- #2 Search: “Breast Neoplasms”[Mesh] Sort by: Most Recent
- #3 Search: ((((((((((Breast Neoplasm[Title/Abstract]) OR (Breast Tumors[Title/Abstract])) OR (Breast Cancer[Title/Abstract])) OR (Mammary Cancer[Title/Abstract])) OR (Malignant Neoplasm of Breast[Title/Abstract])) OR (Malignant Tumor of Breast[Title/Abstract])) OR (Cancer of Breast[Title/Abstract])) OR (Cancer of the Breast[Title/Abstract])) OR (Mammary Carcinoma, Human[Title/Abstract])) OR (Mammary Neoplasms, Human[Title/Abstract])) OR (Breast Carcinoma[Title/Abstract]) Sort by: Publication Date
- #4 Search: #2 OR #3
- #5 Search: “COVID-19”[Mesh] Sort by: Most Recent
- #6 Search: (((((((((((COVID 19[Title/Abstract]) OR (COVID-19 Virus Disease[Title/Abstract])) OR (COVID-19 Virus Infection[Title/Abstract])) OR (2019-nCoV Infection[Title/Abstract])) OR (Coronavirus Disease-19[Title/Abstract])) OR (2019 Novel Coronavirus Disease[Title/Abstract])) OR (2019 Novel Coronavirus Infection[Title/Abstract])) OR (2019-nCoV Disease[Title/Abstract])) OR (Coronavirus Disease 2019[Title/Abstract])) OR (SARS Coronavirus 2 Infection[Title/Abstract])) OR (SARS-CoV-2 Infection[Title/Abstract])) OR (COVID-19 Pandemic[Title/Abstract]) Sort by: Publication Date
- #7 Search: #5 OR #6
- #8 Search: ((randomized controlled trial[Title/Abstract]) OR (controlled clinical trials, randomized[Title/Abstract])) OR (RCT[Title/Abstract]) Sort by: Publication Date
- #9 Search: #1 AND #4 AND #7 AND #8

### 2.4 Study selection

**Figure 1:**
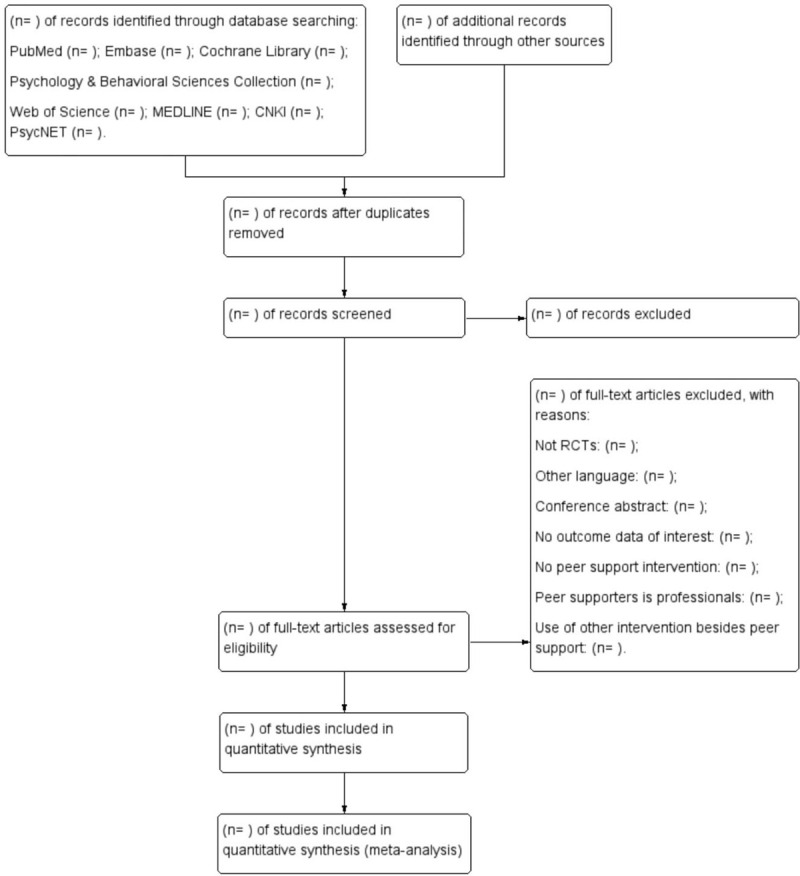
Flow diagram of the literature screening process and results.

Relevant abstracts and titles for all studies will be screened and evaluated by 2 independent reviewers against predefined inclusion criteria, then duplicates or ineligible articles will be excluded based on relevant reasons. The third investigator will resolve any disagreement between the 2 reviewers. The process of screening selection is shown in Figure 1.

### 2.5 Data collection and management

Two reviewers will independently collect data from all studies based on the data extraction form. The following study information will be recorded: first author, year of publication, country, sample size, participant age, recruitment site, remote peer support intervention duration and mode, outcome measures, specific treatment for the control groups, follow-up time and intervention content. The third investigator will resolve any disagreement between the 2 reviewers.

### 2.6 Dealing with missing data

The authors were contacted to obtain missing or unclear data for further analyses. If it fails, we will analyze it based on available data.

### 2.7 Assessment of risk of bias

We will use the tool recommended by the Cochrane Handbook Version 5.1.0^[25]^ to analyze the risk of bias in the trials from the following seven aspects: random sequence generation, allocation concealment, blinding of participants and personnel, blinding of outcome assessment, incomplete outcome data, selective reporting, and other bias. Every item will be classified as “Yes” (low risk of bias), “No” (high risk of bias), or “Unclear” (moderate risk of bias). Disagreements in bias classification will be resolved by discussions among the 2 reviewers and, if necessary, through discussions with the authors.

### 2.8 Assessment of quality of evidence

The evidence evaluation of all results will be summarized by the suggested assessment, development and assessment (GRADE) method.^[26]^ The level of evidence will be divided into high, moderate, low, and very low quality.

### 2.9 Measure of treatment effect

If the evaluated trials used different scales to measure the same outcomes, data will be synthesized by using Hedge g of standardized mean difference (SMD) with 95% confidence interval.

### 2.10 Assessment of heterogeneity

According to the Cochrane Handbook, Chi-Squared test and *I*^2^ value could be used to evaluate the heterogeneity. *I*^2^ values of 25%, 50%, and 75% are considered as low, moderate, and high heterogeneity, respectively.

### 2.11 Assessment of reporting bias

We will perform funnel plots and visually examine the signs of asymmetry to investigate publication bias, then use Egger test^[27]^ as a formal test of publication bias when the number of the included studies was more than 10 (n ≥ 10).

### 2.12 Data synthesis

We will enter group means, standard deviations, and the number of participants in review manager software 5.3 and conducted a random-effects model meta-analysis, and Stata14.0 will be used for sensitivity analysis and Egger test.

### 2.13 Subgroup analysis

If available, we will conduct subgroup analysis based on different intervention modes, different intervention times, different ages of participants, different countries, and outcome measures.

### 2.14 Sensitivity analysis

We will use sensitivity analysis to examine the stability of the results by removing individual trials to determine whether the removed study had a particular effect.

### 2.15 Ethics and dissemination

This work does not require relevant ethical review because there is no data linked to individual patient or animal information. Our research results will be shared and demonstrated through peer-reviewed journals.

## 3 Discussion

Breast cancer is a serious social and public problem. With the development of medical technology, the cure rate of breast cancer has also increased,^[28,29]^ but the stigma generated after breast cancer surgery will lead to anxiety, depression and other mental health problems, affecting the quality of life of patients.^[8,9]^ Remote peer support is a powerful, sustained and affordable form of intervention that has shown unique advantages at this particular time of the COVID-19 pandemic.^[18]^ Research has shown that providing emotional and informational support through peer mentoring and education support has a positive effect on the reduction of stigma in breast cancer survivors.^[30]^ However, there is still no meta-analysis on the effectiveness of remote peer support on stigma in patients after breast cancer surgery. In this paper, we will systematically evaluate whether remote peer support has a positive effect on stigma in patients after breast cancer surgery.

We hope that this study will provide evidence for remote peer support to reduce stigma and improve mental health in patients after breast cancer surgery during the COVID-19 pandemic. In addition, personalized interventions can be developed according to the physical and mental characteristics of patients after breast cancer surgery to maximize the role of remote peer support.

## 4 Conclusion

The purpose of this study was to evaluate the effect of remote peer support on stigma, depression and anxiety in patients after breast cancer surgery during the COVID-19 pandemic. The results may offer hope for reducing mental health problems and improving quality of life in patients after breast cancer surgery.

## Author contributions

**Conceptualization:** Dandan Liang.

**Data curation:** Dandan Liang.

**Formal analysis:** Ruiying Jia, Zhen Wu.

**Methodology:** Dandan Liang, Ruiying Jia, Jingfen Yu, Zhen Wu.

**Project administration:** Chaoran Chen, Guangli Lu.

**Resources:** Dandan Liang, Ruiying Jia.

**Software:** Dandan Liang, Ruiying Jia, Zhen Wu.

**Visualization:** Jingfen Yu.

**Writing – original draft:** Dandan Liang, Guangli Lu.

**Writing – review & editing:** Chaoran Chen.

Ruiying Jia made the same contribution as the first author in the whole process of the study.
